# Evolutionary Pathways in Brain Morphology, Development, and Behavior in the Primate Lineage

**DOI:** 10.1159/bbe/abqag008

**Published:** 2026-09-21

**Authors:** Pasquale Raia, Marina Melchionna

**Affiliations:** Dip. to DiSTAR, Università di Napoli Federico II, Napoli, Italy

**Keywords:** Brain evolution, Brain, Brain size, Brain evolution, Brain-behavior correlation, Brain development, Brain architecture, Brain endocasts, Cortex, Primates

## Introduction

The evolutionary history of the primate brain has long been viewed through the simplistic lens of absolute encephalization and uniform scaling [1]. However, the contributions in this special volume of *Brain, Behavior and Evolution* demonstrate that the evolutionary trajectory leading to modern human cognitive capacities is a highly complex, multi-dimensional process, that is now accessible thanks to new statistical techniques and computational tools. Brain evolution is characterized by modular reorganizations, regional integration, and metabolic trade-offs rather than just a simple, linear increase in volume. By integrating diverse methodologies such as three-dimensional geometric morphometrics, virtual fossil reconstruction, comparative genomics, developmental psychology, and in vivo neuromechanical recording, the papers in this collection look at the paleoneurology and evolutionary neuroscience of primates from an original and uniquely wide perspective. This editorial synthesizes these papers sweeping from macroevolutionary scaling laws across amniotes down to the specific, active behavioral feedback loops that shaped the human brain.

### Macroevolutionary Dynamics of Primate Brain Evolution

The vertebrate brain evolution is governed by broad comparative scaling baselines [2]. Sansalone et al. [3] investigate the macroevolutionary dynamics of brain composition across 201 amniote species representing Squamata, Testudines, Aves, and Mammalia. By applying phylogenetic ridge regression (RRphylo, [4]) and Bayesian phylogenetic regressions [5] to log-transformed brain mass, body mass, and total neuron counts, they demonstrate that major shifts in the rate of neuronal count evolution are exceedingly rare and especially old. Significant positive rate shifts are restricted to highly encephalized clades within birds and mammals such as Galloanserae (fowls), and Boreoeutheria (primates, ungulates, carnivores, and their allies). In contrast, ectothermic vertebrates such as Testudines (turtles and tortoises) and Squamata (lizards, snakes, and their allies) exhibit pronounced negative rate shifts, indicating a conservative pattern of neuron count evolution where species of varying body and brain sizes maintain a low, relatively uniform number of neurons. Pairwise comparisons of regression slopes confirm that Primates display the steepest scaling relationship of neuronal count versus body size, suggesting that the expansion of the primate brain was driven by intense, coordinated selection pressures for increased number of neurons. These findings indicate that a fundamental decoupling of ancestral scaling regimes is an emergent property of highly encephalized clades and, possibly, a necessary precursor for the development of advanced cognitive abilities [2, 3, 6].

While primate evolution is generally characterized by volumetric brain expansion, the comparative record also reveals rare but evolutionarily significant episodes of secondary neocortical reduction or de-encephalization. Aristide [7] explores this phenomenon along the ancestral branch leading to the Callitrichinae (marmosets and tamarins) and the *Aotus* (owl monkey) clade of New World monkeys. Using a Bayesian phylogenetic trait modeling framework (fabric-regression) [8] with medulla volume as a somatic scaling covariate, the author reconstructs a statistically significant reduction in relative neocortex volume along this ancestral platyrrhine branch. To uncover the molecular mechanisms of this reduction, a genome-wide scan was conducted across 18,177 orthologous protein-coding genes. Interestingly, the scan identified 12 genes under positive selection and two under intensified selection. Yet, unlike studies of brain enlargement in the clade, which have reported enrichment of brain-related and neurodevelopmental pathways, no such functional enrichment was detected. Instead, the selective signals were heavily concentrated in genes associated with amino acid and retinol metabolism, coagulation, and mitochondrial biology. The only neurodevelopmental candidate identified was ZNF512B, which interacts with the NuRD chromatin-remodeling complex to coordinate neural-specific transcriptional programs. This genomic landscape suggests that relative neocortical reduction in this lineage did not arise from direct selection on neural phenotypes, but rather emerged as an indirect metabolic by-product of selection on other life-history traits, such as phyletic dwarfism, rapid life cycles, and reproductive specializations [7].

Complementing the macroevolutionary scaling of the neocortex, the primate cerebellum has emerged as a critical site of modular evolution supporting complex behavior. DeMay-Pouliot et al. [9] examine cerebellar lobular expansion across 51 primate species using phylogenetic comparative methods to test whether behavioral convergence is reflected in common neural modifications. By using the developmental boundary that separates the earlier maturing, motor-related anterior lobe from the later-maturing posterior lobe, the authors isolate primarily motor-projecting regions (the anterior lobe and lobule HVI) from prefrontal-projecting structures (Crus I and Crus II) and ask whether these regions evolved on independent trajectories. The phylogenetic analysis reveals that Crus I, which is functionally coupled with the anterior dorsolateral prefrontal cortex and posterior parietal cortex, underwent parallel evolutionary expansion in cognitively complex platyrrhines (Cebinae and Atelinae) and independently in cercopithecoids, apes, and humans. Specifically, Crus I shows a roughly twofold expansion in Cebinae and Atelinae relative to the ancestral platyrrhine baseline. This magnitude matches that seen independently in cercopithecoids, with even greater and nested expansions occurring later in apes and again in humans. In contrast, the motor-related lobule HVI shows generalized anthropoid expansion without convergent platyrrhine-catarrhine patterns. Crucially, the relative size of Crus I explains one half of the variance in species-level general intelligence and ∼80% of the variation in self-control. These findings indicate that cognitive convergence in primates depends on the targeted expansion of prefronto-cerebellar loops, which automate complex mental models and free cortical resources for flexible decision-making during social and ecological interactions [9].

These broad comparative baselines must be integrated with the local structural dynamics that organize the modern human brain. Zeppilli et al. [10] apply three-dimensional geometric morphometrics to a forensic sample of 115 adult human endocasts of documented age and sex to investigate patterns of global and local morphological integration. The cranial endocast serves as a vital paleoneurological proxy, representing the imprint of dural, vascular, and cortical structures [11]. By defining five bilateral anatomical modules corresponding to the frontal, parietal, temporal, occipital, and cerebellar regions, the authors perform module covariance analyses. The results demonstrate a high degree of integration across the entire endocranial surface, with the strongest covariation observed between the frontoparietal modules and between the occipital and cerebellar modules. While sexual dimorphism primarily influences overall endocranial volume without altering shape, aging is significantly associated with regional shape changes. Mass-univariate regressions show that age-related variations are heavily localized along the mid-sagittal profile, particularly from the frontal crest to the superior sagittal sinus groove, which likely reflects modifications of the meningeal-venous interface rather than localized brain atrophy [10].

Within this integrated endocranial architecture, the human parietal lobe stands out as a primary locus of evolutionary modification. Bruner [12] synthesizes 20 years of research on the morphology, development, and evolution of the precuneus and superior parietal lobule. The superior parietal lobule is conventionally divided into an external lateral region and a deeper medial region hidden in the interhemispheric fissure, known as the precuneus. While humans and nonhuman primates share a similar cytoarchitectonic arrangement of these regions, modern humans possess a disproportionately expanded precuneus compared to apes and extinct hominins [12, 13]. The precuneus serves as a central functional hub for visuospatial integration, mental imaging, and the construction of the cognitive self [14]. High-resolution shape analyses reveal that the precuneus exhibits outstanding individual variability among modern adults, with its vertical and longitudinal proportions governed by independent, non-correlated developmental factors. This individual variation is already fully established at birth. However, because of its extreme expansion, the human precuneus is positioned in a complicated topological environment with high physical and vascular constraints, functioning as a watershed zone supplied only by the terminal branches of the anterior and posterior cerebral arteries. These metabolic and vascular limitations render the precuneus highly vulnerable to structural atrophy and neurodegeneration during normal aging and Alzheimer’s disease [12].

### Paleoneurology and Endocast Lateralization

The morphological transformations documented in comparative studies are directly recorded in the hominin fossil record. Di Vincenzo et al. [15] utilize virtual anthropology to examine the virtual endocast of the 400,000-year-old Middle Pleistocene calvaria from Ceprano, Italy. Despite living at a time when derived, Neanderthal-like lineages were already established in Europe, the Ceprano calvaria retains a remarkably plesiomorphic morphology. The digital reconstruction settles its endocranial capacity to 1,165.0 mL, which is consistent with the ancestral state of *Homo heidelbergensis*. Geometric morphometric analyses using a landmark configuration of archaic and modern hominins reveal that Ceprano exhibits a mosaic morphology. Its endocast scores low on the first principal component, aligning with early *Homo erectus* in retaining a long, low, and flattened vault profile. However, its overall capacity is substantially enlarged, and it exhibits early signs of fronto-parieto-temporal expansion. Taphonomic sensitivity analyses on both unrestored and retrodeformed models demonstrate that while right-sided geological compression artificially inflated parietal width, the mosaic signal is biologically genuine. This unique combination supports the following: (i) Ceprano as one of the best candidate to represent the ancestral morphology of *Homo heidelbergensis* [16]; (ii) the crucial position of this *taxon* in the evolution of the genus *Homo* [17]; (iii) a model of mosaic encephalization where absolute brain volume expansion preceded the final structural and cranial globularity that characterizes modern *Homo sapiens* [15].

This volumetric expansion was accompanied by a rapid acceleration in the rate of hemispheric lateralization. Melchionna et al. [18] investigate the macroevolutionary patterns of endocast shape asymmetry across a large sample of 161 endocasts representing 81 living and extinct catarrhine species. Using Phylogenetic Ridge Regression [4] and mesh-area rate mapping [19] the authors map evolutionary rates of asymmetric shape change directly onto the cortical surface. The analysis reveals that the hominin lineage experienced exceptionally high rates of asymmetric structural reorganization compared to all other Old-World monkeys and apes. This lateralization is characterized by a significant leftward expansion of cortical areas associated manipulation and language networks in modern humans. This rapid expansion is widely distributed across the left hemisphere, topologically corresponding to the inferior frontal gyrus (Broca’s area), the ventrolateral prefrontal cortex, the sensorimotor cortex, the supramarginal gyrus, the lateral occipital complex, and the posterior temporal cortex. This left-lateralized network is critical for the integration of sensory and motor information, sequential fine motor planning, and the hierarchical syntactic ordering required for both precise tool crafting and spoken language, providing an evolutionary link between manual praxis and linguistic communication [18].

### Ontogenetic Maturation and Behavioral Scaffolding

The evolutionary transitions documented in fossil endocasts are realized in living individuals through ontogenetic development. Falk [20] explores this interface from an evolutionary developmental perspective, arguing that locomotor transitions in early hominins were a primary driver of cognitive evolution. By transitioning away from the knuckle-walking stages typical of African apes, early hominins introduced new developmental milestones such as hands-and-knees crawling. This crawling phase led progressively to use of freed forelimbs for manual exploration and object manipulation, initiating a developmental cascade of sensory, motor, and cognitive feedback loops that accelerated childhood learning. Central to this theory is the prenatal rhythmic experience hypothesis, which proposes that exposure to a mother’s steady, bipedal footsteps during bipedal walking establishes a steady rhythmic stimulation for the fetus. This maternal walking experience is hypothesized to lay the neurological foundation for auditory-motor entrainment, a cognitive capacity that is unique to humans among primates and served as a vital precursor for the subsequent evolution of language, speech rhythm, and music [20].

Postnatal brain development in modern humans follows a highly structured, developmental schedule that correlates with cognitive milestones [21]. Melchionna et al. [22] map the pace of endocranial shape change during growth by analyzing a cross-sectional sample of 248 digital endocasts ranging from individuals aged 0–18 years. Using evolutionary rate mapping [23] applied to chronological age stages, the authors identify discrete, statistically significant fluctuations in the rate of endocranial shape ontogeny. These structural transitions align precisely with the chronological transitions of Jean Piaget's stages of cognitive development [24]. The analysis reveals a rapid phase of growth across all lobes peaking at age 2, which corresponds to the transition from the sensorimotor to the preoperational stage and supports rapid language acquisition. This is followed by a massive, highly significant peak in shape change velocity 7 years of age, which is driven by localized expansion in the parietal and occipital-cerebellar regions, matching the peak of gray matter volume and the onset of the concrete operational stage. A third peak occurs at age 11, involving localized frontal and parietal reorganizations that align with the transition to the formal operational stage. Widespread area contraction is observed in the frontal and parietal lobes around age 15 years, providing quantitative evidence of synaptic pruning and cortical thinning. In contrast, the temporal lobe is the last to mature, showing late expansion peaks between 13 and 17. The success of this morphometric approach on modern endocasts validates its utility for reconstructing the ontogeny of intelligence in fossil human species where only juvenile crania survive [22].

These developmental capacities are ultimately translated into active, physical expertise through behavioral feedback loops. Eteson et al. [25] investigate the neuromechanical foundations of early stone tool use by simultaneously recording brain activity using electroencephalography (EEG) and muscle recruitment using surface electromyography in subjects performing Oldowan-style flake cutting and hammerstone nut-cracking. During the critical preparatory motor planning phase, expert toolmakers exhibit a highly specialized neural signature, characterized by pronounced beta event-related desynchronization in the left-frontal and premotor regions. This neural activation is coupled with significantly reduced muscle activation in both the dominant and nondominant hands, demonstrating a highly efficient “mind over muscle” neuro-biomechanical coordination. In contrast, novice participants exhibit diffuse parietal activation, reflecting a heavy reliance on raw visuospatial processing, coupled with high, inefficient muscular effort. These results demonstrate that the cognitive and physical demands of intentional tool production required complex motor sequencing and executive planning, suggesting that the emergence of Early Stone Age technologies acted as a major evolutionary pressure that actively drove the structural and functional reorganization of the hominin brain [25].

## Conclusions

The papers in this special volume present a unified, multi-disciplinary framework for understanding how primates and humans acquired their unique cognitive systems. By moving beyond simple measurements of brain volume, these studies demonstrate that encephalization is a mosaic process, where macroevolutionary scaling laws, parallel cerebellar expansions, cortical organization, and local structural integrations establish a flexible physical blueprint [26–29]. This blueprint is subsequently shaped by prenatal and postnatal ontogenetic pacing, locomotor milestones, and the active neuromechanical demands of technology [30–32]. Together, these contributions bridge the gap between hard anatomical structures and complex behavioral outcomes, paving the way for future research in evolutionary neurobiology and virtual anthropology.

## Acknowledgment

We are grateful to BBE editorial team for inviting us to curate the article collection.

## Conflict of Interest Statement

The authors declare no conflict of interest.

## Funding Sources

The authors declare no funding was obtained or used for this study.

## Author Contributions

P.R. and M.M. conceived and wrote the paper together.
